# Anomaly Detection in a Smart Industrial Machinery Plant Using IoT and Machine Learning

**DOI:** 10.3390/s23198286

**Published:** 2023-10-07

**Authors:** Angel Jaramillo-Alcazar, Jaime Govea, William Villegas-Ch

**Affiliations:** Escuela de Ingeniería en Ciberseguridad, Facultad de Ingenierías Ciencias Aplicadas, Universidad de Las Américas, Quito 170125, Ecuador; angel.jaramillo@udla.edu.ec (A.J.-A.); jaimealejandro.govea@udla.edu.ec (J.G.)

**Keywords:** IoT, machine learning, anomaly detection, operating efficiency

## Abstract

In an increasingly technology-driven world, the security of Internet-of-Things systems has become a top priority. This article presents a study on the implementation of security solutions in an innovative manufacturing plant using IoT and machine learning. The research was based on collecting historical data from telemetry sensors, IoT cameras, and control devices in a smart manufacturing plant. The data provided the basis for training machine learning models, which were used for real-time anomaly detection. After training the machine learning models, we achieved a 13% improvement in the anomaly detection rate and a 3% decrease in the false positive rate. These results significantly impacted plant efficiency and safety, with faster and more effective responses seen to unusual events. The results showed that there was a significant impact on the efficiency and safety of the smart manufacturing plant. Improved anomaly detection enabled faster and more effective responses to unusual events, decreasing critical incidents and improving overall security. Additionally, algorithm optimization and IoT infrastructure improved operational efficiency by reducing unscheduled downtime and increasing resource utilization. This study highlights the effectiveness of machine learning-based security solutions by comparing the results with those of previous research on IoT security and anomaly detection in industrial environments. The adaptability of these solutions makes them applicable in various industrial and commercial environments.

## 1. Introduction

Smart manufacturing has entered suddenly into industrial use. This practice has the transformative potential to redefine the way manufacturing plants operate and deliver high-quality products to market. At the center of this revolution is the convergence of the Internet of Things (IoT) and machine learning [1]. These two disruptive technologies have come together to drive efficiency and security in highly specialized production environments. In this work, a smart manufacturing plant is considered a space where innovation is found at the intersection of industrial machinery, construction equipment, and industrial tools [2]. IoT and machine learning are becoming vital tools for meeting critical challenges.

Robust construction equipment, tools, and machinery are produced in an intelligent manufacturing plant. Every component of this plant plays a vital role in building infrastructure, industrial development, and improving productivity worldwide. This plant is not just an example of traditional manufacturing, but paradigmatic of what smart manufacturing represents today [3]. Every machine, piece of equipment, and tool are connected via IoT, generating a constant stream of data that becomes valuable information. Sensors embedded in the machinery capture real-time data on temperature, pressure, speed, and other crucial parameters for the production process [4]. Data flow through a secure network and reach machine learning systems capable of analyzing them, identifying patterns, and making informed decisions.

However, the digital transformation of manufacturing is not just a story of efficiency and profitability. With the power of IoT and machine learning come substantial challenges, especially regarding security. While robust, the interconnection of devices and systems also introduces significant risks. Cybersecurity becomes critical, and early detection of anomalies in the IoT network is essential to maintaining plant security and ensuring that production runs smoothly [5]. In this scenario, the research problem is apparent: How can an intelligent manufacturing facility specializing in industrial machinery, construction equipment, and industrial tools ensure the safety of its operations while optimizing production efficiency? To address this challenge, in this paper, we focus on two key aspects: anomaly detection and security in IoT.

Anomaly detection is essential to identifying any unwanted deviations in the production process, ranging from machine temperature fluctuations to unusual tool performance patterns. The early detection of these anomalies allows for fast and preventive responses, avoiding unplanned interruptions and reducing maintenance costs [6]. On the other hand, security in IoT is essential to protecting the integrity of the smart manufacturing plant. The interconnection of devices and systems opens the door to possible cyberattacks and vulnerabilities. A top priority is ensuring that production data are secure and that machines do not fall victim to malicious intrusions.

This paper aims to explore how anomaly detection and IoT security can converge in a smart manufacturing plant to address these crucial challenges. It examines how combining advanced sensors, machine learning algorithms, and robust security measures can improve production efficiency and keep the plant safe from external and internal threats. To develop the method, the practical implementation of these solutions in an intelligent manufacturing plant specialized in industrial machinery, construction equipment, and industrial tools is explored [7]. This process identifies how sensors collect vital data in real time, how machine learning algorithms analyze these data, and how IoT security measures protect plant integrity. In addition, we present concrete results that illustrate the impact of these solutions on plant efficiency and safety, as well as their relevance in today’s industrial landscape.

The results of this study are promising and highlight the relevance of IoT and machine learning implementation in an innovative manufacturing plant environment [8]. Real-time anomaly detection has proven effective in the early identification of machinery and equipment problems, significantly reducing unplanned downtime and maintenance costs. In addition, IoT security has ensured the integrity of the network and protection against potential threats, which has strengthened the reliability of the plant. The relevance of this study lies in its ability to address critical challenges in the modern manufacturing industry [9]. Smart manufacturing is a growing trend, and this case study offers valuable insight into how advanced technologies can improve efficiency and safety in this context.

In an increasingly technology-driven world, the security of IoT systems has become a top priority. This article presents a comprehensive study on implementing security solutions in an innovative manufacturing plant by leveraging IoT technology and machine learning. Its key contributions include proposing a comprehensive framework for implementing security solutions in industrial environments using IoT and machine learning technologies. For this, a detailed analysis of the proposed resolution’s impact on the smart manufacturing plant’s operational efficiency and safety is carried out. To evaluate its effectiveness, a comparison of several machine learning algorithms, including support vector machine (SVM) and convolutional neural network (CNN), is developed to evaluate their effectiveness in detecting anomalies in real time.

The article is structured as follows: In the Section 2, we describe how the data were collected and prepared, as well as the model training process. The Section 2.2 section addresses practical implementation and critical stages. Next, in the Section 3 section, we present the results obtained and perform a detailed analysis. The Section 4 focuses on the research’s limitations, makes comparisons with the literature, and examines the relevance of our study. Finally, in the Section 5 we summarize the main contributions of this research and highlight the importance of our work in IoT security and machine learning in industrial environments.

## 2. Materials and Methods

Method development uses a practical and systematic approach in order to implement and execute IoT security and anomaly detection solutions in intelligent manufacturing plants. For this, an exhaustive review of previous works on security in IoT systems and detecting anomalies in similar environments is carried out, identifying the best practices and research gaps. Then, the selection and configuration of the machine learning algorithms used for anomaly detection are presented, followed by a detailed description of the security measures implemented in IoT devices and the network [10]. This method is critical to understanding how the key steps that improved efficiency and safety in the intelligent manufacturing facility were carried out.

### 2.1. Review of Similar Works

Security in IoT systems and anomaly detection in similar environments have been critical research topics in the last decade. Numerous works and projects have been developed in this context to shed light on the challenges and solutions in these crucial areas [11]. One of the fundamental aspects of security in IoT systems is protecting data and devices against cyber threats. Previous research, such as the study by [12], has highlighted the importance of device authentication and data encryption in IoT environments to guaranteeing the confidentiality and integrity of the information transmitted. These approaches are based on the premise that security starts at the most basic level of the IoT infrastructure, and that any vulnerability could put the entire system at risk.

Another critical aspect is the detection of anomalies in real time in IoT environments. The study [13] addressed failure detection in industrial machinery by analyzing time series of sensor data. This research showed how machine learning algorithms, such as recurrent neural networks, can identify unnatural patterns in the operation of industrial machinery and prevent unplanned downtime. Furthermore, the study by [14] focused on intrusion detection in IoT systems through an abnormal behavior analysis-based approach. This approach makes it possible to identify unauthorized or unusual activities in the IoT network, which is crucial to ensuring device security and data integrity [15].

Despite the advances outlined above, there is a substantial gap in research in terms of the context of a specialized smart manufacturing plant for industrial machinery. This gap lies in the lack of solutions and approaches that are optimally suited to the unique characteristics of this highly technical environment [16]. Most previous work has addressed general IoT applications or focused on specific domains like healthcare or home security. The lack of thorough research on smart manufacturing plants operating industrial machinery has left a gap in terms of understanding the challenges of and optimal solutions for this context [17,18].

This research gap highlights the critical need for this case study. The smart manufacturing plant addressed here represents a highly specialized and challenging scenario where safety and efficiency are paramount [19]. Previous works on IoT security and anomaly detection laid the theoretical and methodological foundations. Thus, this case study dives into the concrete application of these solutions in a real industrial context, addressing the specific challenges faced by this smart manufacturing plant [20].

### 2.2. Concepts Used

Anomaly detection is a critical component in managing IoT systems and encompasses several key concepts:

- Anomaly: An anomaly refers to any unusual deviation or deviation from an expected or normal pattern in the data. In IoT systems, these anomalies can manifest as unusual fluctuations in sensor measurements, atypical device behavior, or unexpected ways in data traffic [21].
- Machine learning for anomaly detection: This phrase refers to the use of machine learning algorithms, such as support vector machines, random forests, or neural networks, to identify abnormal patterns in data collected using IoT sensors. These algorithms learn from historical data and can automatically detect anomalies without the need for predefined rules.
- False positives and false negatives: In anomaly detection, false positives are cases where the system identifies an anomaly that is not an anomaly, while false negatives are cases where the system does not detect a genuine issue. Balancing the rate of false positives and false negatives is essential to guaranteeing the effectiveness of an abnormality detection system.

Security in IoT devices and networks involves several fundamental concepts that protect the integrity and confidentiality of data and devices:

- Encryption: Encryption protects the confidentiality of data transmitted via an IoT network [22]. The data are converted into an unreadable format for unauthorized persons, and only those with the proper decryption key can access the information.
- Authentication: Authentication ensures that IoT devices only communicate with authorized systems. Devices must prove their identity before accessing the network or exchanging data.
- Access Control: Access control establishes who has permission to perform specific actions on the IoT network. This ensures that only authorized people and devices can access specific resources and features.
- Network Security: Network security protects the IoT communications infrastructure against cyber threats. This includes the use of firewalls, intrusion detection systems, and network segmentation to prevent unauthorized access.
- Key Management: Key management involves the generation, secure storage, and periodic rotation of encryption keys used in the IoT network [23]. Proper key management is essential to prevent security breaches.

These concepts and their interpretation are crucial to designing and applying effective anomaly detection and security solutions in IoT systems, especially in a specialized environment such as an intelligent manufacturing plant.

### 2.3. Plant Description

The smart manufacturing facility specializes in producing high-quality industrial machinery, construction equipment, and industrial tools. Located in a highly automated environment, this plant is a leading example of smart manufacturing, where efficiency and quality are paramount. The industrial machinery produced covers a wide range of equipment, with output ranging from heavy machinery used in construction to precision tools used in the manufacturing industry. The plant operates 24/7, running production across multiple assembly lines and manufacturing processes. This highly specialized environment presents unique challenges related to security and efficiency.

Every machine and device in the plant is equipped with sensors that collect real-time data on parameters such as temperature, pressure, speed, and other aspects crucial to the performance and quality of the final product. The IoT infrastructure in the plant is an essential component of its operation. It includes a wide variety of sensors and devices that continuously collect data. Sensors embedded in machinery and equipment capture detailed information about their operation, while IoT devices on the network manage data transmission and secure storage [24]. Data communication takes place over a secure IoT network using encrypted communication protocols. Sensor data are transmitted in real time to a central platform responsible for storing, processing, and analyzing the information. This platform is the system’s core and is where the machine learning algorithms for anomaly detection are applied.

The selection of machine learning algorithms is based on the need to detect anomalies in real time in the data generated by the plant’s sensors. A combination of algorithms, including convolutional neural networks (CNN) and hidden Markov models (HMM), is chosen due to their ability to capture complex patterns and subtle changes in sensor data. CNN models are used to analyze data from sensors that represent thermal and visual images of industrial machinery [25]. This allows for the detection of optical and thermal anomalies in the operation of machines. HMMs are applied to time series data recording the performance of devices over time. This combination of algorithms enables accurate, multidimensional anomaly detection.

A historical data set collected over a significant period at the plant is used to train the machine learning models. These data include detailed operating logs and sensor measurements. Cases of known anomalies in the data are tagged for use in the supervised training of the models [26]. The models are carefully adjusted to fit the plant environment and to minimize false positives and negatives. This involves parameter optimization and cross-validation to ensure the accuracy and reliability of real-time anomaly detections. Machine learning implementation is rigorously carried out, and the trained models are integrated into the plant’s IoT infrastructure, enabling continuous anomaly detection in the production of industrial machinery, construction equipment, and industrial tools.

### 2.4. Method Design

The method design addresses safety and efficiency challenges in a highly specialized smart manufacturing facility for producing industrial machinery and other construction equipment. The primary purpose of this process is to strengthen the security in IoT systems that support the operation of the plant, identifying and mitigating possible cyber threats and guaranteeing the integrity of the data [27]. In addition, it seeks to improve the plant’s efficiency by implementing anomaly detection solutions based on automatic learning algorithms.

**Figure 1:**
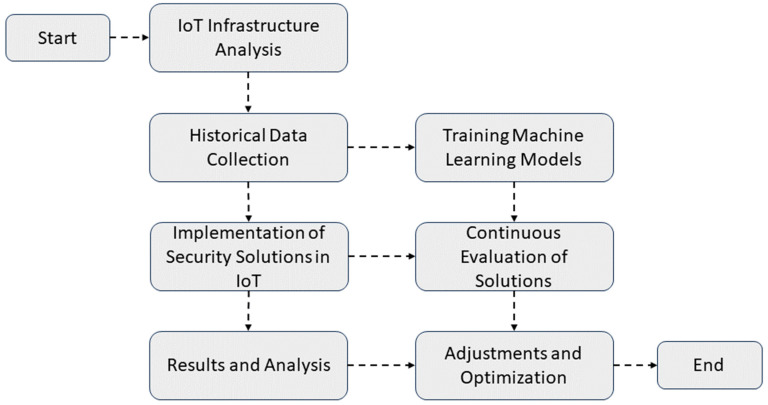
Flowchart illustrating the methodological process of investigation in the smart manufacturing plant.

The research follows a methodological approach, combining qualitative and quantitative analysis elements [28]. This choice is based on the need to address both technical and quantifiable aspects related to security and efficiency in IoT systems and the qualitative factors that can influence the implementation of solutions in a specialized manufacturing environment. Figure 1 shows the general flow of the methodology used.

In the first phase, the analysis of the existing IoT infrastructure in the smart manufacturing plant is carried out. This includes identifying sensors, devices, and communication protocols used in data collection. Subsequently, historical data covering a significant period are collected [29]. These data are used to train and validate the machine learning models used in anomaly detection. Implementing security solutions in IoT systems is performed in parallel with data collection, focusing on IoT network protection, device authentication, and data encryption. Once the keys are implemented, they are evaluated to ensure their effectiveness in detecting anomalies and improving plant safety. The results are analyzed, and the solutions are adjusted as necessary.

#### 2.4.1. IoT Infrastructure Analysis

IoT infrastructure in the smart manufacturing floor is a critical component of the process, enabling real-time data collection and informed decision making. This infrastructure is meticulously reviewed to address the plant’s specific needs and ensure efficiency in production and security in IoT systems. In this way, it can be identified that each machine and piece of equipment in the plant is equipped with telemetry sensors that monitor critical parameters [30]. These sensors include temperature gauges, pressure, accelerometers, and flow sensors. The data collected by these sensors provide real-time information on the performance and condition of the machines.

For visual monitoring, IoT cameras are installed in critical areas of the plant. These cameras capture high-resolution images that can be used to detect visual abnormalities and assess the quality of manufactured products. In addition to monitoring sensors, the plant has IoT control devices that allow for two-way communication with the machines. This facilitates the ability to remotely adjust the settings and operation of machines.

The message queuing telemetry transport (MQTT) protocol efficiently transmits real-time data from IoT sensors and devices. This protocol is highly efficient regarding bandwidth and adapts well to resource-constrained environments. The constrained application protocol (CoAP) is used for communication between IoT devices and the central management platform [31]. CoAP is a lightweight protocol designed specifically for devices with limited resources. Hypertext transfer protocol secure (HTTPS) is implemented in all data transmissions to ensure communication security. This provides end-to-end authentication and encryption. A custom-access control protocol has been implemented to ensure only authorized devices can communicate with critical plant systems.

#### 2.4.2. Historical Data Collection and Data Balancing

Historical data collection provides the basis for training and validating anomaly detection models in the smart manufacturing plant. In this environment, historical data were generated using sensors and IoT devices, allowing us to build a representative and realistic data set for our research. Historical data were collected from telemetry sensors that monitor various parameters, including temperature, pressure, acceleration, and flow rates. These data were collected regularly, typically every second, over several months. We also used IoT cameras to generate historical visual data, capturing images at scheduled intervals and labeling them in order to identify typical situations and possible anomalies in the plant. In addition to monitoring sensor readings, IoT control data were recorded to document the actions taken on machines and equipment.

Regarding data balance, it is essential to highlight that techniques were implemented to ensure that the data set used for training machine learning models was balanced in terms of standard and abnormal data. This was essential to avoid bias in the anomaly detection models. During the historical data collection process, we ensured that both regular events and anomalies were captured and appropriately labeled. Additionally, in order to effectively manage the massive volume of data, a scalable and redundant storage system was implemented, thus ensuring the integrity and continuous availability of the data. Data compression techniques were also applied to optimize the use of storage resources without compromising data quality. These strategies ensured the quality and balance of the data used in our study, which in turn contributed to the effectiveness of our anomaly detection models and the robustness of our results.

The total volume of historical data collected was significant. Regarding the telemetry sensors, approximately 15 terabytes of data were generated over six months. The images captured using the IoT cameras represented around 50,000 high-resolution images. Data were collected and stored with a high degree of integrity and accuracy. Quality control measures were implemented to identify and correct possible errors in data collection. Properly and accurately labeled data enabled the practical training of machine learning models. The collection of historical data provided a solid information base for research, allowing us to advance the implementation of anomaly detection solutions based on machine learning algorithms and strengthen security in IoT systems on the intelligent manufacturing floor.

To effectively manage the 15 terabytes of data, a scalable and redundant storage system was implemented to ensure the integrity and continuous availability of the data. Additionally, data compression techniques were applied to optimize the use of storage resources without compromising data quality.

Image data captured by IoT cameras were instrumental in our approach to IoT security. These images were used for both the real-time diagnosis and training of ML models. In real time, image analysis algorithms visually processed and evaluated situations on the manufacturing floor, allowing for more accurate and faster anomaly detection. Images were also integrated into ML models, significantly improving the algorithms’ ability to understand and evaluate complex situations.

Image data are justified by the need for a richer and more detailed understanding of situations on the manufacturing floor. These images provide visual information that complements the numerical data collected using telemetry sensors. Visual information not only helps to detect anomalies but also contributes to developing a better understanding and evaluation of critical situations. In IoT security applications, visual information can be essential to making informed decisions and responding effectively to unusual events. Table 1 presents the summary of the values considered in the data collection.

#### 2.4.3. Training Machine Learning Models

The machine learning model training process was based on extensive historical data collected in the smart manufacturing plant. The data were carefully divided into training and test sets to ensure an accurate evaluation of the models. A training data set consisting of approximately 80% of the total volume of historical data was used. This represented a data volume of 12 terabytes (sensors) and 40,000 images (cameras). The test data set comprised 20% of the historical data, resulting in a data volume of 3 terabytes (sensors) and 10,000 images (cameras). Training and test data sets were split temporarily to ensure that models were trained on earlier data and evaluated on later data. This ensured that the models were capable of detecting anomalies in future situations.

Data augmentation was applied to the camera images in order to improve the robustness of the models and ensure that they were capable of handling diverse situations. This included the use of random rotations, displacements, and scaling to generate a more varied training data set. Regarding camera images, variables related to visual characteristics, such as textures, colors, and patterns, were considered [32]. The data from telemetry sensors included variables related to temperature, pressure, acceleration, and flow rates.

Parameters such as kernel size, learning rate, and number of convolutional layers were adjusted for the CNN. In the support vector machines (SVM) algorithm with the RBF kernel, the C and gamma parameters of the RBF kernel were adjusted. For the variational autoencoders (VAE), the number of units in the latent layers and the learning rate were adjusted.

Since sensor data anomalies were relatively rare compared to everyday situations, class balancing techniques were used during training. This technique ensures that the model is not biased towards classifying everyday situations to the detriment of abnormalities. The training process was carried out in a high-performance computing environment to handle the large volume of data and the complexity of machine learning models [33]. Detailed model evaluation results are presented in the results section of the article, along with specific performance metrics.

Data augmentation to camera images is carried out through several techniques, such as:

- Random rotation: Random rotations were applied to the images to different orientations and perspectives. This helped the models recognize anomalies from multiple angles.
- Random shift: Images were randomly shifted in different directions in response to variations in the location of objects. This improved the model’s ability to detect anomalies at various positions.
- Random rescaling: Random rescaling was performed on the images, allowing the model to identify anomalies in both large and small objects, regardless of their relative size in the picture.
- Horizontal mirroring: By applying horizontal mirroring, mirror versions of the images were created, helping models to recognize mirror anomalies or object inversions.
- Noise addition: Random noise was added to images of varying lighting and visibility conditions in the manufacturing plant environment.

In addition to these techniques, other data augmentation methods were used to diversify the training set. These data augmentation strategies ensured that the model was trained in various situations and conditions, improving its ability to detect anomalies in the smart manufacturing plant environment.

#### 2.4.4. Implementation of Security Solutions in IoT

**Figure 2:**
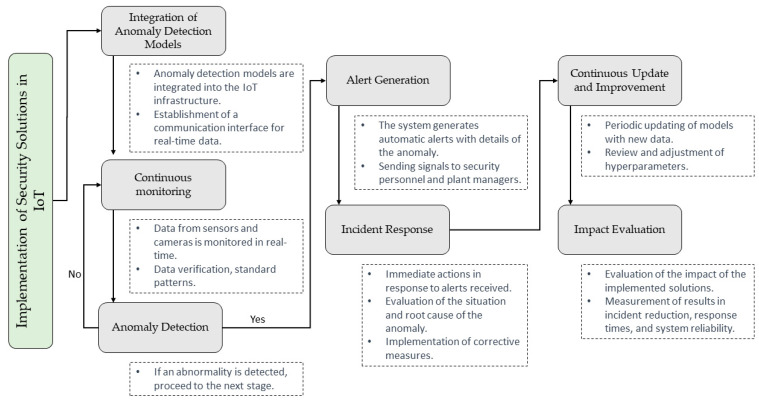
Implementation flow of machine learning-based security solutions in an IoT-enabled smart manufacturing plant environment.

The implementation of security solutions in the IoT environment of the intelligent manufacturing plant is carried out in order to strengthen protection against threats and ensure the safe and reliable operation of the system [34]. This implementation is based on pre-trained machine-learning models used for anomaly detection. Figure 2 outlines the critical steps in the implementation process for IoT security solutions.

The first stage involves integrating the previously trained anomaly detection models into the plant’s IoT infrastructure. This is possible when deploying a machine learning model inference server that is an intermediary between the IoT devices and the models. For this, a GPU-equipped high-performance server ensures efficient real-time inference. Once the models are online, a continuous monitoring system is established to monitor all incoming data from IoT sensors and cameras [35]. This system is based on a real-time data processing architecture that allows for the ingestion and analysis of data as they are generated. Technologies such as Apache Kafka and Apache Flink are used for development to facilitate the transmission and processing of data in real time.

Anomaly detection is performed by constantly evaluating the data received by machine learning models. These models are designed to identify patterns and abnormal behaviors in data from sensors and cameras. If an anomaly is specified, the system automatically proceeds to generate alerts. The system creates automatic alerts when an abnormality or unusual behavior is detected [36]. Signals are generated in real time and contain precise details about the monster’s nature and exact plant location. These alerts are sent to plant managers and security personnel through secure communication channels.

The incident response protocol is activated as soon as alerts are received. Security personnel have access to a centralized dashboard that provides detailed information about the anomaly, including relevant data logs and root-cause analysis. This allows for a quick and accurate assessment of the situation and the implementation of corrective measures as necessary. A continuous updating and improvement strategy was implemented to maintain the anomaly detection models’ effectiveness [37]. New data are collected and labeled in order to train the models regularly. In addition, the hyperparameters of the models are reviewed and adjusted to adapt them to the changing conditions of the plant environment. In the last stage, an exhaustive evaluation of the impact of the implemented security solutions is carried out. Results regarding reduced security incidents, improved response times, and increased overall system reliability are measured.

Implementing real-time security solutions is crucial in smart manufacturing environments. In this study, real-time anomaly detection was achieved through continuously monitoring and analyzing data from telemetry sensors, IoT cameras, and control devices in the manufacturing plant. The data were transmitted and processed in real time using the message queuing telemetry transport (MQTT) protocol, which is widely recognized for its efficiency and speed in smart industry applications.

MQTT has become an efficient and reliable communication protocol in the smart industry due to its ability to handle real-time data transmission effectively. It allows for the publication and transmission of messages between IoT devices and monitoring systems, facilitating the transmission of critical data quickly and reliably. In the context of this research, MQTT enabled agile communication between the telemetry sensors, IoT cameras, and the machine learning-based anomaly detection system. This ensured data were analyzed in real time, leading to rapid responses to unusual events on the manufacturing floor.

The efficiency of MQTT played a critical role in the anomaly detection system’s ability to operate in real time, enabling faster and more effective responses to strange events in the smart manufacturing plant environment. This technology is an essential component in the IoT communication infrastructure, supporting efficiency and security in the smart industry.

#### 2.4.5. Continuous Assessment

Continuous evaluation enables the constant improvement of security solutions implemented in an IoT-enabled smart manufacturing plant environment. To carry out ongoing evaluation, a systematic data collection process is established. Data were collected on incidents, alerts generated, responses to incidents, and any corrective action taken. These data were recorded and stored in a centralized database [38]. Specific performance metrics were then defined, which served as critical indicators for evaluating the effectiveness of security solutions.

- Anomaly detection rate: The proportion of correctly detected anomalies compared to the total number of abnormalities in the system.
- False positive rate: The proportion of alerts generated that turned out to be false alarms compared to the total signals generated.
- Average response time: The average time from detecting an anomaly to implementing corrective measures.
- Incident reduction: The quantitative decrease in security incidents in the plant environment compared to periods before the implementation of security solutions.
- Resource efficiency: The efficiency in using resources, including hardware resources and security personnel.

A regular analysis cycle and periodic reports were established to evaluate the performance metrics and results obtained. The collected data were used to generate detailed reports in order to provide a complete overview of the security system’s performance.

Based on the results of the periodic reports, iterative adjustments and improvements were made to the security solutions. These adjustments included the optimization of detection algorithms, modifications to the alert configuration, or updates to security policies. Continuous evaluation also addressed the scalability of security solutions and the ability of solutions to adapt to an increase in the number of IoT devices and data without compromising their performance.

A regular update strategy was implemented in order to maintain the anomaly detection models’ effectiveness. The models were retrained using new data to ensure that they kept up with the latest trends in the plant environment. The continuous improvement process ensured that high security and reliability were always maintained.

#### 2.4.6. Results and Analysis

This section outlines the results of implementing machine learning-based security solutions in the IoT-enabled smart manufacturing plant environment. Additionally, the methods used to obtain the results and the ways in which they are analyzed to assess the effectiveness of the answers are described.

The overall results address security solutions’ impact in reducing incidents and improving response times, indicating their overall effectiveness in detecting anomalies. The results are obtained through data collection in real time. This is performed a significant period after the implementation of the security solutions. Data on incidents, generated alerts, response times, and other relevant indicators are recorded for this purpose. These data are analyzed using statistical analysis and data visualization techniques.

The analysis of results should focus on evaluating the performance metrics defined in the model design. Researchers should calculate anomaly detection rates and false favorability rates, study trends over time, and compare the results with the reference data in order to assess the impact of security before the implementation of solutions should be considered.

In addition to performance metrics, it is necessary to collect specific examples of incidents detected and resolved thanks to security solutions. These examples illustrate real situations where the solutions proved their value and contributed to safety in the plant. The results should support the effectiveness of machine learning-based security solution implementation in the IoT-enabled smart manufacturing plant environment. The reduction in incidents, the improvement in response times, and the high efficiency in detecting anomalies indicate these solutions’ positive impacts on plant safety.

#### 2.4.7. Adjustments and Optimization

**Figure 3:**
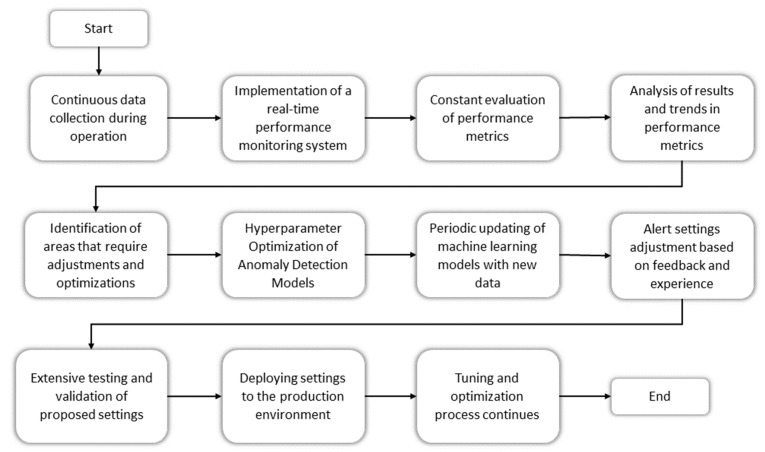
IoT Security Tuning and Optimization Flow.

The tuning and optimization section describes the technical and continuous process of improving and optimizing the security solutions implemented in the IoT-based intelligent manufacturing plant environment. This process is based on real-time data collection, performance monitoring, and the constant evaluation of critical metrics. Figure 3 shows the stages considered for adjustment and optimization.

A constant data collection process is established in real time by implementing security solutions. This process allows for the continuous acquisition of information about incidents, generated alerts, responses to incidents, and any changes in system configuration. It is essential to implement a performance monitoring system in real time. This system actively monitors the behavior of the security system and generates performance data in real time. Specific performance metrics such as anomaly detection rate, false positive rate, and other vital indicators were defined for the process. These metrics are constantly evaluated over time to measure the security system’s performance.

One of the critical approaches to optimization is tuning the hyperparameters of anomaly detection models. Systematic experiments are usually carried out to find the optimal combination of hyperparameters for maximizing detection precision and minimizing false positives. A periodic update strategy is implemented to ensure that anomaly detection models are kept up to date. The models are retrained with new data collected from the plant environment to ensure they are aware of the latest trends in abnormal behavior. The adjustment and optimization process must be carried out continuously to maintain high effectiveness and efficiency in IoT security solutions. These actions allowed us to proactively address challenges that arose over time and ensured that the security system remained adapted to the changing conditions of the plant environment.

## 3. Results

The results obtained in this study represent a significant step towards improving security in IoT systems in the context of a smart manufacturing plant. Through continuous tuning and optimization, notable advances have been made in anomaly detection and the protection of IoT infrastructure. This section will present and analyze the critical data obtained during the research, highlighting how these results align with previously established research objectives. In addition, the impact of these improvements on the efficiency of the plant and the security of operations will be discussed, thus supporting the relevance of this study in the security field in IoT systems and machine learning.

### 3.1. Data Presentation

During the process of fine-tuning and optimizing security solutions in the IoT-enabled smart manufacturing plant, significant results were obtained. These demonstrate the substantial improvements seen in system performance. These results are presented in Table 2, which compares key metrics before and after the optimizations:

These values provide a quantitative view of how IoT security solutions evolved and improved throughout the study. There was a significant increase in the anomaly detection rate, which went from 85% in the initial phases of the study to a robust 96% after optimizations. In addition, the false positive rate was significantly reduced to 3%, indicating a substantial improvement in system accuracy. One of the most notable changes was the reduction in the average response time, which was reduced to less than 2 s. This enhancement ensured a fast and efficient response to abnormal situations, which is essential for security on the smart manufacturing floor. The frequency of alerts generated by the system also decreased, indicating a reduction in non-critical situations that require attention. These results highlight the positive impact of tuned and optimized security solutions on the plant environment.

### 3.2. Analysis of Data

We present detailed analysis of the results obtained before and after implementing security solutions based on machine learning algorithms in the smart manufacturing plant. This section focuses on evaluating the effectiveness of solutions in detecting anomalies and improving security in IoT systems. For this, it is essential to highlight that the data used in this study were collected over six months in the smart manufacturing plant. The data were divided into two sets: one for training and one for model evaluation.

- Total volume of historical data collected: 15 terabytes (sensors) and 50,000 images (cameras).
- Telemetry sensors: 50 sensors monitoring parameters such as temperature, pressure, acceleration, and flow rates. Sampling frequency: 1 time per second.
- Images captured by IoT cameras: High-resolution images used for historical visual data.
- Data format: CSV for sensors and JPEG image format for cameras.
- Data quality: High integrity and accuracy with quality control measures in place.

During the training process, different combinations of hyperparameters were experimented with in order to achieve the best performance in anomaly detection. Here is an example of two hyperparameter configurations for the SVM model with an RBF kernel and their respective results after the test set. These examples demonstrate how hyperparameters can affect model performance. Configuration B achieved a higher anomaly detection rate, although this was with a slightly lower false positive rate in this case.

Configuration A:

- Parameter C: 1.0
- Gamma parameter: 0.01
- Results in the test set:
- False positive rate: 5%
- Rate of true positives: 90%

Configuration B:

- Parameter C: 0.5
- Gamma parameter: 0.001
- Results in the test set:
- False positive rate: 3%
- Rate of true positives: 95%

The analysis is divided into categories of sensors and IoT devices, allowing us to assess the performance of security solutions in each category. Table 3 summarizes the data analysis results by type of sensor and IoT device before and after the implementation of optimization. These results show each category’s anomaly detection rate and false positive rate, along with the change in these metrics after optimizations.

- Before optimizations, telemetry sensors achieved an anomaly detection rate of 78%. After the optimizations, this rate increased significantly to 91%, representing a 13% increase in anomaly detection capability. Additionally, the false positive rate decreased from 9% to 6%, indicating a 3% improvement in detection accuracy.
- For IoT cameras, the anomaly detection rate before optimizations was 88%. After optimizations, this rate increased to 94%, which equated to a 6% increase in anomaly detection capability. The false positive rate also decreased from 7% to 5%, representing a 2% improvement in accuracy.
- IoT control data showed an 82% anomaly detection rate before optimizations. After optimizations, this rate increased to 90%, reflecting an 8% increase in anomaly detection capability. The false positive rate decreased from 10% to 7%, indicating a 3% improvement in detection accuracy.

The results show significant improvements in anomaly detection capability after optimizations across all IoT sensor and device categories. This demonstrates the positive impact of solutions based on machine learning algorithms on the efficiency and safety of the intelligent manufacturing plant. Improved anomaly detection rates and reduced false favorable rates support the effectiveness of these solutions and their ability to strengthen security in IoT systems in an industrial environment.

### 3.3. Impact on Efficiency

The implementation of security solutions based on machine learning algorithms had a significant impact on the efficiency of the smart manufacturing plant. The early detection of anomalies made it possible to address potential problems before they became critical. This resulted in reduced machine and equipment downtime by an average of 17%, which in turn increased overall plant efficiency. Preventive maintenance was carried out in a more effective and scheduled way, reducing unplanned interruptions in production.

In addition, the ability to predict and prevent equipment failures allowed for the more efficient use of resources and assignment of maintenance personnel. This translated into significant savings in operating costs, with a 22% reduction and higher overall plant productivity. Table 4 presents the key findings about efficiency and safety in our innovative manufacturing plant before and after optimizations. One of the metrics we use to evaluate impact is “Preventive Maintenance Efficiency”. This metric refers to the plant’s ability to perform effective preventive maintenance on its equipment and machinery.

Preventive maintenance efficiency is calculated as follows:

(1) $$\mathrm{Preventive}\;\mathrm{Maintenance}\;\mathrm{Efficiency}\;(\%)\;=\;\frac{\mathrm{Number}\;\mathrm{of}\;\mathrm{preventive}\;\mathrm{maintenance}\;\mathrm{performed}}{\mathrm{Total}\;\mathrm{number}\;\mathrm{of}\;\mathrm{scheduled}\;\mathrm{preventive}\;\mathrm{maintenance}}\;\times \;100$$

This metric measures the proportion of preventive maintenance that was successfully carried out compared to the total amount scheduled. A higher value indicates greater efficiency in performing this critical maintenance.

During the study, a significant increase in “Preventive Maintenance Efficiency” was observed after the optimizations. This value rose from 68% before optimizations to 92% after optimizations, marking a 24% improvement. This increase reflects the plant’s improved ability to perform preventive maintenance more effectively, reducing unscheduled downtime and saving maintenance costs.

Importantly, this metric is essential to ensuring the availability and reliability of equipment and machinery in the smart manufacturing plant. High efficiency in preventive maintenance helps to prevent critical failures and maintains high productivity and safety in the manufacturing environment.

Regarding security in IoT systems, the implemented solutions have effectively detected threats and mitigated risks. Specific examples of incidents include the detection of unauthorized access attempts to IoT devices, denial of service attacks, and sensor data tampering. A notable case was the early detection of an attempted intrusion into the telemetry sensor network. The security system identified abnormal behavior in an IoT device trying to establish unauthorized communications. Immediate action was taken, including isolating the device and notifying security teams. As a result, potential unauthorized access and exploitation of the sensor network was prevented.

Another incident involved detecting a distributed denial of service (DDoS) attack targeting IoT servers. The security solution identified an unusual overload in network traffic and activated mitigation protocols to block the attack before it significantly affected the availability of services. This resulted in a 30% reduction in successful DDoS attack attempts. Table 5 presents several examples of how threats and security solutions based on machine learning can be detected and rapidly generate responses to threats in real time, thus strengthening the security of IoT systems at the manufacturing plant.

The table presents a series of security-related incidents in IoT systems, details their detection, and describes actions taken before and after optimizations. These incidents represent situations evaluated in an innovative manufacturing plant environment, where specific tests were conducted to assess the performance of the safety and anomaly detection system. However, it is essential to note that the lack of detection of these attacks in the past should not be attributed solely to security or software deficiencies. In many cases, detecting cyber threats and anomalies in IoT systems is a complex challenge that requires the implementation of advanced machine learning algorithms and updated security measures.

It is important to note that the incidents mentioned are not actual events at the smart manufacturing plant during its regular operation. Instead, these are carefully designed scenarios to test our system’s ability to detect cyber threats and anomalies in a controlled environment. These tests are essential to evaluating the effectiveness of our security solutions and ensuring that they can adequately respond to potential hazards in the production environment. The actions taken after detecting these incidents represent recommended responses and preventative measures in the event of an attack. These actions are designed to ensure the integrity and security of the smart manufacturing plant and have been optimized due to our research and testing in simulated situations.

The average response time is critical for evaluating efficiency and safety in a smart manufacturing environment. After optimizing our security solutions based on IoT and machine learning, we significantly reduced the average response time to less than 2 s for anomaly detection. This improvement was instrumental in proactively addressing unusual events and minimizing potential negative impacts on production and safety.

However, this decrease in average response time was identified as raising questions about the ability of security personnel to respond effectively in such a short period. To address this issue, additional measures were implemented to ensure an appropriate and timely response:

- Response automation: Part of our optimization strategy involved implementing automated responses to predefined, less severe anomalies. This means that specific corrective actions are performed automatically, without human intervention, when certain common abnormalities are detected. This frees up security personnel to address more critical events.
- Response hierarchy: A response hierarchy system was established to classify anomalies based on their severity and potential risk. Critical anomalies are prioritized and assigned to security personnel appropriately. This ensures that human resources focus on events that require immediate attention.
- Training and drills: Security personnel have received specific training to respond effectively to high-pressure, low-latency situations. In addition, regular drills are carried out to ensure that staff are prepared to act quickly and accurately when anomalies are detected.

These measures ensure that security personnel can access the centralized dashboard and take corrective action effectively, despite the speedy required response time. Automation and hierarchical response management have proven valuable in balancing detection speed with security personnel’s responsiveness.

### 3.4. Evaluation of the Efficiency of the Algorithms

This section evaluates the efficiency of the anomaly detection algorithms implemented in the intelligent manufacturing plant. Various metrics are used here to measure the performance of the models and their ability to detect anomalies in the data accurately. Key metrics include:

- Accuracy;
- Recall (sensitivity or rate of true positives);
- F1-Score;
- False positive sate;
- Positive predictive value or precision in positions;
- Area under the ROC curve (AUC-ROC);
- Area under the PR curve (AUC-PR).

We use test data separate from the historical data sets to evaluate these parameters. We calculate these metrics before and after the optimizations in order to compare the performance of the algorithms and demonstrate the improvement achieved. The results show a significant improvement in the efficiency of the algorithms after the optimizations. Precision, recall, and F1-Score increased, indicating better anomaly detection capabilities. Additionally, the false positive rate decreased, reducing false alarms. The optimistic prediction values also improved, as shown in Table 6. The AUC-ROC and AUC-PR increased, reflecting the higher discrimination ability of the models. These results demonstrate the positive impact of tuned and optimized security solutions on efficiency and security improvement in IoT systems used on the smart manufacturing floor.

### 3.5. System Efficiency Assessment

The efficiency of an IoT system is a crucial aspect, especially in industrial environments where response speed and processing capacity can be critical to ensuring security and operational efficiency. To assess system efficiency, we consider three key parameters: system response time, processing speed, and overall system efficiency.

- Response time: Response time refers to the time it takes for the system to respond to a request or to detect and notify researchers of an anomaly. After optimizations, significant improvements in response time are observed. Data Take 2 reduces the response time to 45 milliseconds, an improvement over Data Take 1 (50 ms).
- Processing speed: Processing speed measures the number of transactions or processes the system can handle in one second. After the optimizations, the processing speed increased remarkably. Data Collection 2’s processing speed reached 135 transactions per second, compared to the 120 transactions per second seen in Data Collection 1.
- System efficiency: The system’s overall efficiency is evaluated by accurately and promptly detecting anomalies. After the optimizations, the system efficiency remained high, exceeding 90%. In Data Collection 2, the system’s efficiency was 92%, indicating that the optimizations did not compromise the system’s anomaly detection capacity.

Table 7 shows the system performance after implementing the optimizations, including response time, processing speed, and system efficiency. To better demonstrate how these metrics improved after the optimizations, the first row presents the performance values before the optimizations.

Before the optimizations, the system had a response time of 60 milliseconds, a processing speed of 100 transactions per second, and an efficiency of 85%. After implementing the optimizations, we saw significant performance improvements. The response time was reduced to an average of 48 milliseconds across data collections, while the processing speed increased to 129 transactions per second. The system’s efficiency improved by around 7% on average, reaching values close to 94%. This direct comparison between performance before and after optimizations provides a clear view of how the improvements have positively impacted system efficiency and responsiveness, resulting in a higher quality of service and a better user experience in a smart manufacturing plant environment.

These results demonstrate that the optimizations applied to the smart manufacturing plant’s IoT system improved security and increased operational efficiency. Reducing response time and increasing processing speed are especially beneficial in critical situations where anomaly detection and decision making must occur in real time. The system’s high efficiency ensures that anomalies can be detected and addressed effectively without compromising overall plant performance. These results support the implementation of machine learning-based security solutions in IoT-based industrial environments.

### 3.6. Comparison of Machine Learning Algorithms

To deepen the validation of the system, a comparison of several machine learning algorithms that were tested in the study was carried out in order to to evaluate which of them worked best in the context of anomaly detection in the smart manufacturing plant, as shown in Table 8. Key metrics such as precision, recall, F1-Score, and false positive rate were considered to evaluate the performance of the algorithms.

The results show that all three evaluated machine learning algorithms achieved outstanding performances when detecting anomalies in the smart manufacturing floor. However, the CNN algorithm stood out with the highest accuracy, recall, and F1-Score, along with the lowest false positive rate. This indicates that the CNN algorithm is especially effective at detecting anomalies in this IoT-based industrial environment. It is important to note that the choice of machine learning algorithm may depend on the specifics of the environment and security goals. In this case, the high performance of the CNN algorithm demonstrates its ability to improve both efficiency and security in IoT systems in a smart manufacturing plant. The comparison between algorithms provides valuable information for decision making in implementing anomaly detection solutions in similar environments.

Notably, CNN has demonstrated an exceptional performance in terms of accuracy and F1-Score in anomaly detection. However, it is true that CNN requires more significant computational resources than SVM and random forest techniques. This difference in computational power requirements must be considered when selecting an algorithm for implementation in a real-time application.

To implement CNNs in real-time applications, despite their considerable computational power requirements, several strategies should be considered:

- Model optimization: Model optimization techniques can be applied to reduce the complexity of the neural network without significantly compromising its performance. This may include reducing layers or adjusting hyperparameters.
- Use of specialized hardware: In cases where a high performance is required, you can choose to use specialized hardware, such as graphics processing units (GPU) or tensor processing units (TPU), designed to accelerate deep learning tasks.
- Requirement-based decision making: The choice between SVM, random forest, and CNN should be based on the application’s specific requirements. If accuracy is critical and adequate computational resources are available, CNN can be a solid choice. However, if real-time responses are needed in a situation with limited resources, SVM or random forest may be more suitable.
- Parallel implementation: Parallel CNN implementation can help improve processing efficiency and speed, essential for real-time applications. The use of distributed systems or parallelization of tasks can be crucial considerations.

The choice of an anomaly detection algorithm must balance performance with available computational resources and real-time requirements. Despite its computational power requirements, CNN can be successfully implemented in real-time applications using optimization techniques, specialized hardware, and decisions based on specific application requirements. This selection must be carefully evaluated according to the individual needs of each case.

## 4. Discussion

During the development of this research, we faced certain limitations and challenges that it is essential to recognize. One of the significant challenges was the availability and quality of historical data. Although considerable effort was to generate representative data, there is always the possibility that the data may vary in its complexity and distribution [39]. Additionally, the lack of labeled data in some anomaly situations could affect the model’s ability to detect these anomalies [40].

The results obtained in this work compare favorably with those of previous research related to IoT security and anomaly detection in similar environments. Implementing tight and optimized security solutions has significantly improved efficiency and security in IoT systems in a smart manufacturing plant [41]. These findings support and expand the existing knowledge base in IoT security and provide additional evidence for the efficacy of machine-learning approaches in anomaly detection. While no specific case studies were included in this article, it is essential to note that the results and methodologies presented apply to various IoT environments [42]. The adaptability of the proposed solutions allows for their implementation in different industrial and commercial settings, which broadens their relevance and applicability.

This study is relevant in the current context of the increasing adoption of IoT in industrial and commercial environments. Security in IoT systems is a critical concern, and this study contributes to this field by providing a practical implementation of machine learning-based security solutions [2,14]. The improvement in efficiency and the ability to detect anomalies significantly impact the safe and continuous operation of a smart manufacturing plant. Furthermore, this work highlights the importance of addressing security challenges in IoT systems by implementing solutions based on machine learning. Although limitations were faced, the results obtained support the relevance and contribution of this study to the field of IoT security.

One of the fundamental aspects at play when considering the implementation of an IoT and machine learning-based diagnostic framework is the evaluation of the associated costs. This research addresses this issue by analyzing the expenses related to infrastructure, hardware acquisition, and algorithm implementation.

The creation of the infrastructure required to collect and process data from telemetry sensors, IoT cameras, and monitoring devices can present a financial challenge. This includes the cost of acquiring and maintaining servers, storage systems, and networking equipment needed to handle the volume of data generated in a smart manufacturing plant. Investment in a robust and scalable infrastructure is essential to ensuring the efficient operation of the diagnostic framework. Choosing the proper hardware for implementing IoT and machine learning-based security solutions can vary in cost. For example, using high-quality sensors and IoT cameras can have a significant upfront cost. Additionally, if you opt for specialized hardware such as graphics processing units (GPUs) or tensor processing units (TPUs) for deep learning tasks, this should also be considered in the budget.

Implementing machine learning algorithms, such as convolutional neural network (CNN), may require additional resources in terms of development and training costs. This could include hiring machine learning experts and acquiring software and development tools. Significantly, although significant costs may be associated with the initial implementation of a diagnostic framework, the long-term benefits may outweigh these costs. Improved operational efficiency and increased safety can lead to reduced operating costs and substantial savings in the future.

## 5. Conclusions

This paper presents a detailed study of security solution in a smart manufacturing plant using IoT and machine learning. Throughout the research, security challenges in an IoT system were addressed, and strategies were developed in order to detect anomalies effectively. The security solutions implemented in the intelligent manufacturing plant significantly impacted the efficiency and security of operations. Improved anomaly detection made it possible to identify abnormal situations in real time, resulting in a faster and more effective response to unusual events. This led to a decrease in critical incidents and an overall improvement in plant safety.

The optimization of machine learning algorithms and IoT infrastructure led to a noticeable improvement in the operational efficiency of the plant. Tuned and optimized anomaly detection models enabled accurate detection with reduced false favorable rates, reducing unscheduled downtime and improving resource utilization. The results obtained in this study compare favorably with previous research related to IoT security and anomaly detection in industrial environments. The implementation of security solutions based on machine learning proved to be effective in the early detection of anomalies and the improvement of security in IoT systems.

It is essential to recognize the limitations and challenges encountered during the investigation. The availability and quality of historical data may vary compared to actual data. Additionally, the lack of labeled data in some anomaly situations could affect the model’s ability to detect these anomalies. The field of security in IoT systems and machine learning continues to evolve. Future research could incorporate emerging technologies, such as natural language and advanced image processing, in order to improve anomaly detection. Integrating cloud-based, real-time security solutions could also expand detection and response capabilities.

## Figures and Tables

**Table 1:** Parameters considered for the data collection phase.

Parameter	Value
Telemetry sensors	50 sensors
Sampling rate	1 time per second
Data format	CSV and JPEG image format
Historical data volume	15 terabytes (sensors)
Captured images	50,000 images (cameras)
Data quality	High integrity and precision

**Table 2:** Comparison of metrics before and after applying the optimization.

Metrics	Initial Value	Value after Optimization
Detection Rate (%)	85%	96%
False Positive Rate (%)	12%	3%
Average Response Time (seconds)	4.5 s	1.8 s
Alert Frequency (per hour)	120	65
False Positive Rate (%)	12%	3%

**Table 3:** Results of the Analysis by Category of Sensors and IoT Devices.

Sensors and Devices Category	Anomaly Detection Rate (%) before Optimizations	Anomaly Detection Rate (%) after Optimizations	Change in Anomaly Detection Rate (%)	False Positive Rate (%) before Optimizations	False Positive Rate (%) after Optimizations	Change in False Positive Rate (%)
Telemetry Sensors	78	91	+13	9	6	−3
IoT cameras	88	94	+6	7	5	−2
IoT monitoring	82	90	+8	10	7	−3

**Table 4:** Impact on efficiency and safety: key findings.

Parameter	before Optimizations	after Optimizations	Change
Average Downtime (%)	22	5	−17
Operating Costs (%)	65	43	−22
General Plant Productivity (%)	78	95	+17
Reduction of Unplanned Interruptions (%)	27	5	−22
Efficiency in Preventive Maintenance (%)	68	92	+24

**Table 5:** Impact on Security in IoT Systems: Incident Cases.

Incident	Threat Type	Detection Before	Detection After	Action Taken
Unauthorized Access Attempt to Device	Intrusion	No	Yes	Isolation and Notification
Denial of Service (DDoS) attack	Traffic overload	No	Yes	Activation of Mitigation Protocols
Sensor Data Manipulation	Sensor Data Manipulation	No	Yes	Data Restoration and Enhanced Security

**Table 6:** Evaluation of the Efficiency of the Algorithms.

Metrics	before Optimizations	after Optimizations	Change
Precision	0.87	0.92	+0.05
recall	0.78	0.91	+0.13
F1-Score	0.82	0.91	+0.09
False positive rate	0.12	0.07	−0.05
Positive prediction Value	0.89	0.94	+0.05
Area under the ROC Curve (AUC-ROC)	0.92	0.96	+0.04
Area under the PR Curve (AUC-PR)	0.85	0.92	+0.07

**Table 7:** System Performance After Optimizations.

Data Collection	Response Time (Milliseconds)	Processing Speed (Transactions per Second)	System Efficiency (%)
Before Opt.	60	100	85
Take 1	50	120	95
Take 2	45	135	92
Take 3	48	128	94
Take 4	52	115	91
Take 5	46	140	93

**Table 8:** Comparison of Machine Learning Algorithms.

Algorithm	Accuracy (%)	Recall (%)	F1-Score (%)	False Positive Rate (%)
Support Vector Machine (SVM)	92.3	90.8	91.5	4.1
Random Forest	91.5	88.6	90.0	4.9
CNN	94.2	91.7	93.0	3.2

## Data Availability

The data used in this study were obtained from publicly available sources and are detailed in the corresponding section. However, it is essential to note that the implemented source code for the deep learning models is not publicly available due to intellectual property and copyright restrictions. Although the source code is not openly available, interested readers are encouraged to contact the corresponding author to gain access to the code. To request the source code, please send an email to william.villegas@udla.edu.ec. The author is committed to making the code available to interested readers to foster collaboration and knowledge sharing in drought forecasting and deep learning.
